# Supplementary figures and images for: Global longitudinal strain and plasma biomarkers for prognosis in heart failure complicated by diabetes: a prospective observational study
Source: BMC Cardiovasc Disord. 2024 Mar 5;24:141. doi: 10.1186/s12872-024-03810-5 (PMC10913625; doi:10.1186/s12872-024-03810-5)

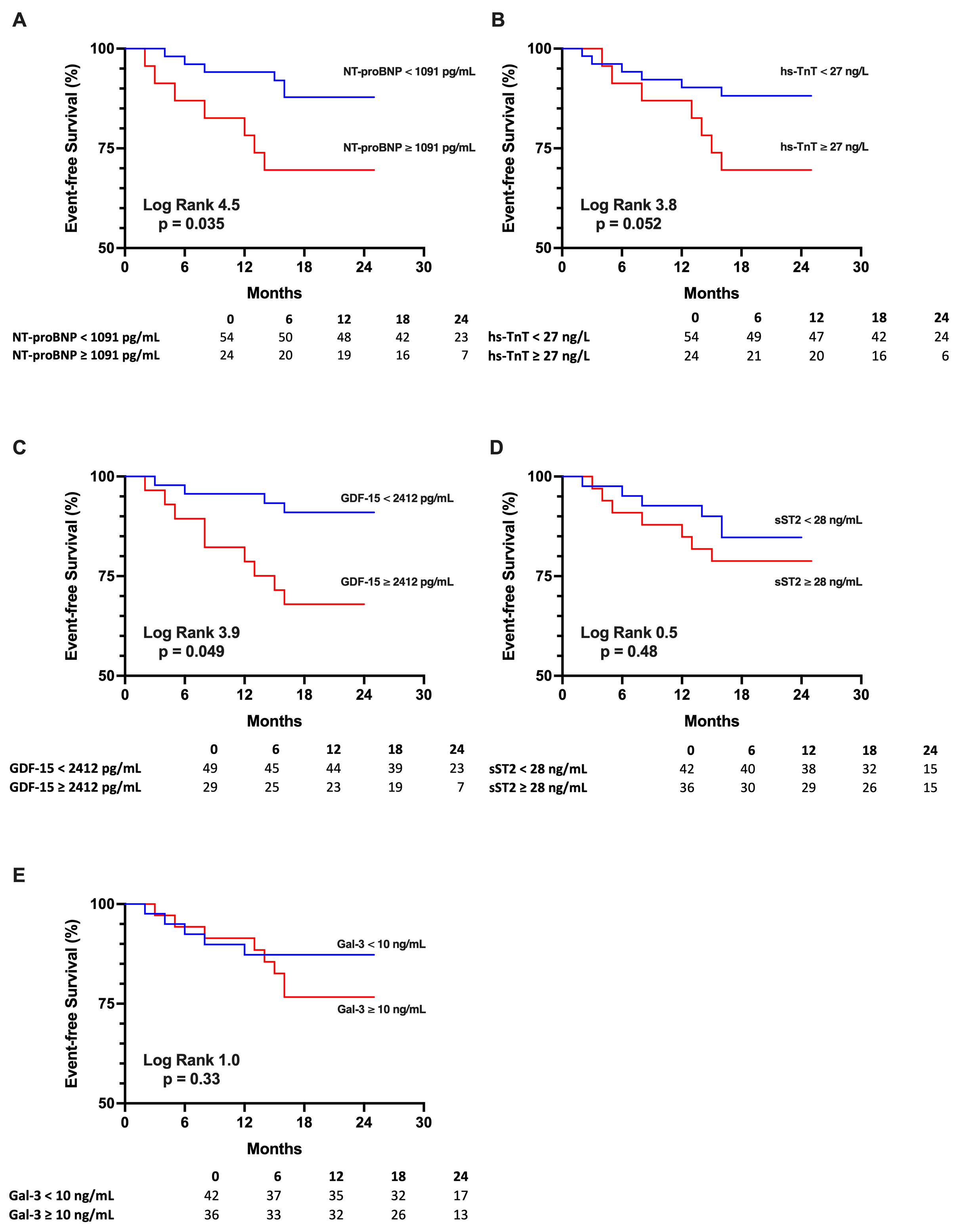

Supplement: Supplementary file 1 — Supplementary Material 1 [file 12872_2024_3810_MOESM1_ESM.tif]

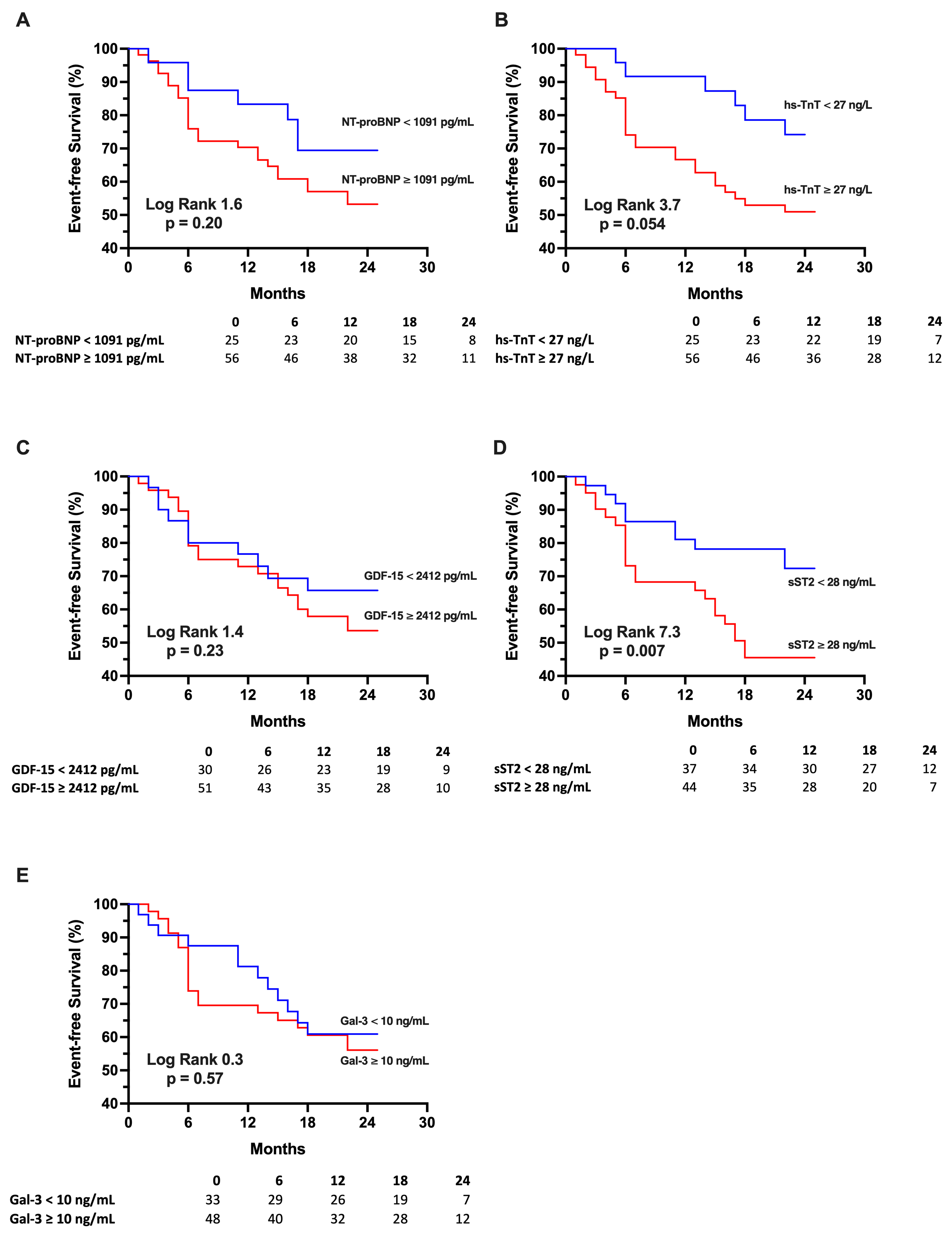

Supplement: Supplementary file 2 — Supplementary Material 2 [file 12872_2024_3810_MOESM2_ESM.tif]
