# Supplementary material for: Global longitudinal strain and plasma biomarkers for prognosis in heart failure complicated by diabetes: a prospective observational study
Source: BMC Cardiovasc Disord. 2024 Mar 5;24:141. doi: 10.1186/s12872-024-03810-5 (PMC10913625; doi:10.1186/s12872-024-03810-5)
Supplement: Supplementary file 3 — Supplementary Material 3 [file 12872_2024_3810_MOESM3_ESM.docx]

**Supplemental Figure Titles and Legends**

**Supplemental Figure 1. Event-free survival curves in patients with diabetes and above median GLS (-9.9%) stratified by:** (A) NT-proBNP; (B) hs-TnT; (C) GDF-15; (D) sST2 and (E) Gal-3. NT-proBNP and GDF-15 demonstrated additional prognostic value in this group. Abbreviations: GLS: global longitudinal strain; NT-proBNP: N-terminal pro-brain natriuretic peptide; hs-TnT: high-sensitivity troponin T; GDF-15: growth differentiation factor 15; sST2: soluble ST2; Gal-3: galectin 3.

**Supplemental Figure 2. Event-free survival curves in patients with diabetes and below median GLS (-9.9%) stratified by:** (A) NT-proBNP; (B) hs-TnT; (C) GDF-15; (D) sST2 and (E) Gal-3. sST2 demonstrated additional prognostic value in this group. Abbreviations: GLS: global longitudinal strain; NT-proBNP: N-terminal pro-brain natriuretic peptide; hs-TnT: high-sensitivity troponin T; GDF-15: growth differentiation factor 15; sST2: soluble ST2; Gal-3: galectin 3.

**Supplemental Tables**

Supplemental Table 1. Rates of LGE according to diabetes status in patients without history of coronary artery disease.

|  | **No Diabetes Mellitus**  **(n=72)** | **Diabetes Mellitus**  **(n=44)** | **p Value** |
| --- | --- | --- | --- |
| LGE Type |  |  |  |
| Nil, n (%) | 39 (54) | 16 (36) | 0.062 |
| Non-ischaemic, n (%) | 24 (33) | 19 (43) | 0.287 |
| Ischaemic, n (%) | 9 (13) | 9 (21) | 0.251 |

**Abbreviations**:; LGE: Late gadolinium enhancement.

Supplemental Table 2. Cause of death according to the presence or absence of diabetes.

|  | **No Diabetes Mellitus**  **(n=156)** | **Diabetes Mellitus**  **(n=159)** |
| --- | --- | --- |
| Cardiovascular, n (%) | 1 (14) | 5 (33) |
| CVA, n (%) | 0 | 1 (7) |
| Respiratory, n (%) | 0 | 2 (13) |
| Bleeding, n (%) | 0 | 2 (13) |
| Undetermined, n (%) | 6 (86) | 5 (33) |

**Abbreviations**:; CVA: Cerebrovascular accident.
